# Supplementary material for: Nanosensitizer-mediated augmentation of sonodynamic therapy efficacy and antitumor immunity
Source: Nat Commun. 2023 Nov 1;14:6973. doi: 10.1038/s41467-023-42509-7 (PMC10620173; doi:10.1038/s41467-023-42509-7)
Supplement: Supplementary file 1 — Supplementary Information [file 41467_2023_42509_MOESM1_ESM.pdf]

Supporting information

Nanosensitizer-mediated augmentation of sonodynamic therapy efficacy and antitumor immunity

Yongjiang Li[1,#], Wei Chen[1,#], Yong Kang[2], Xueyan Zhen[1], Zhuoming Zhou[1], Chuang Liu[1], Shuying Chen[1], Xiangang Huang[1], Hai-Jun Liu[1], Seyoung Koo[1], Na Kong[1,3,4], Xiaoyuan Ji[2,4], Tian Xie[4,5,6,7,*], Wei Tao[1,*]

[1] Center for Nanomedicine and Department of Anesthesiology, Brigham and Women's Hospital, Harvard Medical School, Boston, MA 02115, USA

[2] Academy of Medical Engineering and Translational Medicine, Medical College, Tianjin University, Tianjin, 300072, China

[3] Liangzhu Laboratory, Zhejiang University Medical Center, Hangzhou, China

[4] School of Pharmacy, Hangzhou Normal University, Hangzhou, Zhejiang, 311121, China

[5] Key Laboratory of Elemene Class Anti-Cancer Chinese Medicines, Hangzhou Normal University, Hangzhou, Zhejiang, 311121, China

[6] Engineering Laboratory of Development and Application of Traditional Chinese Medicines, Hangzhou Normal University, Hangzhou, Zhejiang, 311121, China

[7] Collaborative Innovation Center of Traditional Chinese Medicines of Zhejiang Province, Hangzhou Normal University, Hangzhou, Zhejiang, 311121, China

[#] These authors contributed equally to this work: Yongjiang Li, Wei Chen

[*] To whom correspondence may be addressed.

Email: tianxie@hznu.edu.cn (T.X.); wtao@bwh.harvard.edu (W.T.)

Contents

Supplementary Figure 1. Size distribution, zeta potential and dispersibility of SnSNPs@PEG.

Supplementary Figure 2. XPS survey spectrum of SnSNPs.

Supplementary Figure 3. UV−Vis absorbance spectra of the time-dependent oxidation of DPBF (80 μg mL^{-1} in ethanol) by the following treatment: (a) US (1 MHz, 1 W cm^{-2}, 50% duty cycle) only, and (b) SnSNPs@PEG (200 μg mL^{-1}) only.

Supplementary Figure 4. UV−Vis absorbance spectra of the time-dependent oxidation of MB (5 μg mL^{-1} in PBS) by the following treatment: (a) US (1 MHz, 2 W cm^{-2}, 50% duty cycle) only, and (b) H_2O_2 (50 μM) + US.

Supplementary Figure 5. UV−Vis absorbance spectra of the time-dependent oxidation of MB (5 μg mL^{-1} in PBS) by the following treatment: (a) SnSNPs@PEG (100 μg mL^{-1}) + H_2O_2 (50 μM), (b) SnSNPs@PEG only, and (c) SnSNPs@PEG + H_2O_2 + NIR_{808} (1.0 W cm^{-2}).

Supplementary Figure 6. UV−Vis absorbance spectra of the time-dependent oxidation of GSH (30 μg mL^{-1} in PBS) by the following treatment: (a) SnSNPs@PEG (200 μg mL^{-1}), (b) US (1 MHz, 2 W cm^{-2}, 50% duty cycle) only, (c) GSH + NIR_{808} and (d) SnSNPs@PEG + NIR_{808} (1.0 W cm^{-2}).

Supplementary Figure 7. Photothermal performance of SnSNPs@PEG.

Supplementary Figure 8. Relative viability of 4T1 cells after incubation with different concentrations of SnSNPs@PEG for 24 and 48 h.

Supplementary Figure 9. Bright-field microscope images showing time-dependent cellular uptake of SnSNPs@PEG by 4T1 cells.

Supplementary Figure 10. Bright-field microscope images showing 4T1 cells after various treatments.

Supplementary Figure 11. Time-dependent temperature increase profile in 4T1 tumor-bearing mice showing the *in vivo* photothermal effect of SnSNPs@PEG.

Supplementary Figure 12. Comparison of luminescence intensity of 4T1 tumor areas after various treatments.

Supplementary Figure 13. Change in the 4T1 tumor volume after various treatments.

Supplementary Figure 14. Change in body weight of 4T1 tumor-bearing mice during the SnSNPs@PEG-mediated strategy treatment.

Supplementary Figure 15. Spleen of 4T1 tumor-bearing mice after SnSNPs@PEG-mediated treatment strategy.

Supplementary Figure 16. Antitumor efficacy of SnSNPs@PEG-mediated SDT in an orthotopic RIL-175-HCC mouse model.

Supplementary Figure 17. Semi-quantitative analysis of green fluorescence intensity shows the ROS level in tumors after various treatments.

Supplementary Figure 18. Gating strategies to identify $CD45^+CD3^+CD4^+$ and $CD45^+CD3^+CD8^+$ T lymphocytes in the tumor, spleen and lymph nodes of 4T1 tumor-bearing mice following the SnSNPs@PEG-mediated treatment strategy.

Supplementary Figure 19. Gating strategies to identify $CD45^+CD3^+CD4^+$ and $CD45^+CD3^+CD8^+$ T lymphocytes in the RIL-175-HCC following the SnSNPs@PEG-mediated treatment strategy.

Supplementary Figure 20. Comparison of immune cell levels in 4T1 tumors in different groups after SnSNPs@PEG-mediated treatment strategy.

Supplementary Figure 21. Flow cytometry analysis of (a) $CD45^+$ cells, (b)(c) $CD45^+CD3^+$ lymphocytes, $CD45^+CD3^+CD4^+$ T lymphocytes and $CD45^+CD3^+CD8^+$ T lymphocytes in RIL-175-HCC after different treatments (n = 3).

Supplementary Figure 22. Comparison of the levels of (a) $CD45^+$ immune cells, (b) $CD45^+CD3^+$ T cells, (c) $CD45^+CD3^+CD4^+$ T cells, and (d) $CD45^+CD3^+CD8^+$ T cells in RIL-175-HCC-bearing mice after various treatments (n = 3).

Supplementary Figure 23. Comparison of $CD45^+CD3^+CD8^+$ T cell levels in spleens and lymph nodes of 4T1 tumor-bearing mice in different groups after SnSNPs@PEG-mediated treatment strategy.

Supplementary Table 1. Comparison of the bandgap of SnSNPs with other nano-sonosensitizers.

Supplementary Table 2. Antibody used for the flow cytometry analysis of T cells.

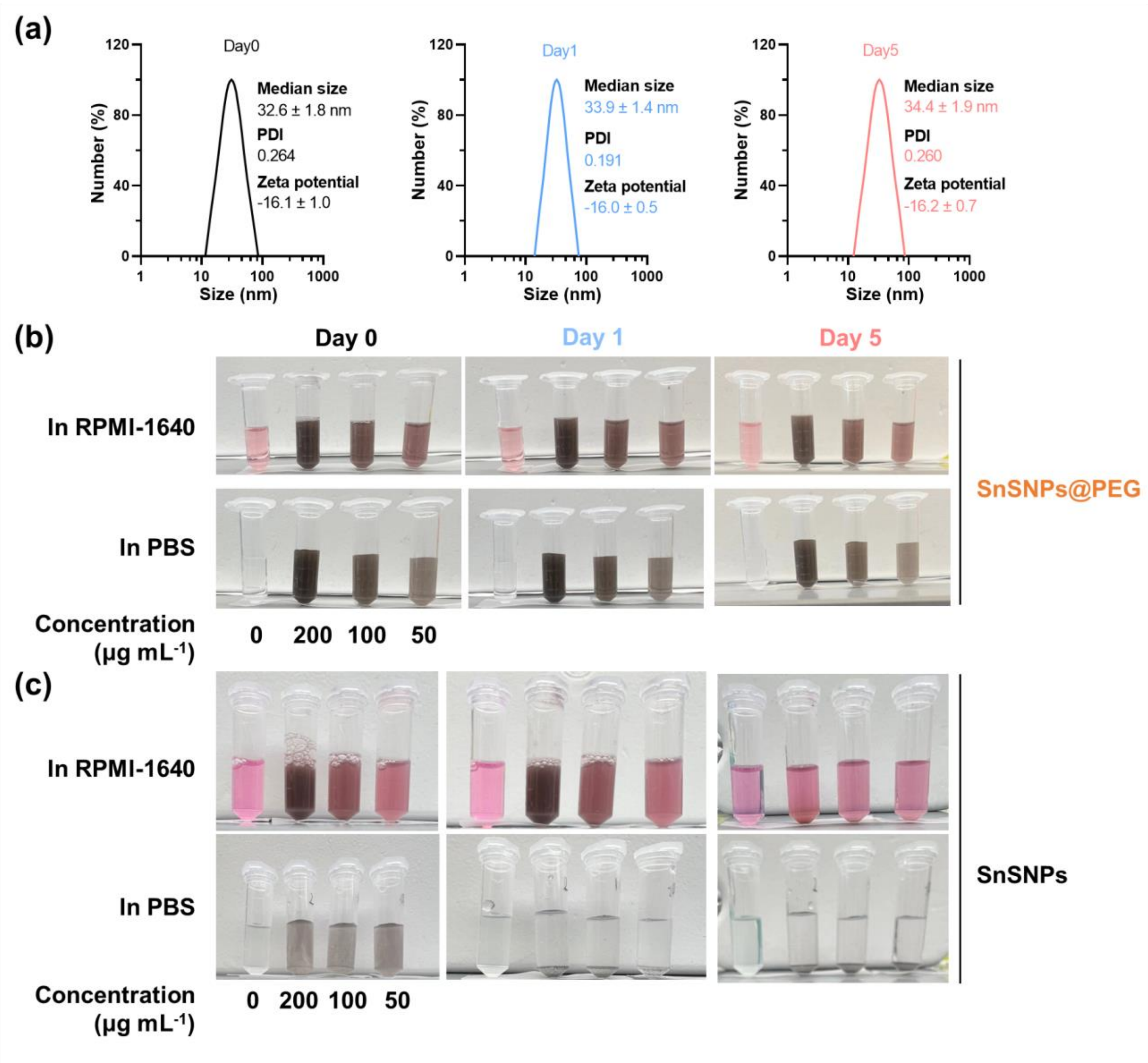

Figure S1. Size distribution, zeta potential and dispersibility of SnSNPs@PEG. a) DLS measurement of size distribution, and zeta potential of SnSNPs@PEG in PBS on different days. b) Photographs showing the dispersion of (b) SnSNPs@PEG and (c) SnSNPs at various concentrations in RPMI-1640 cell culture medium or PBS after incubation at 4 ℃ for different time intervals.

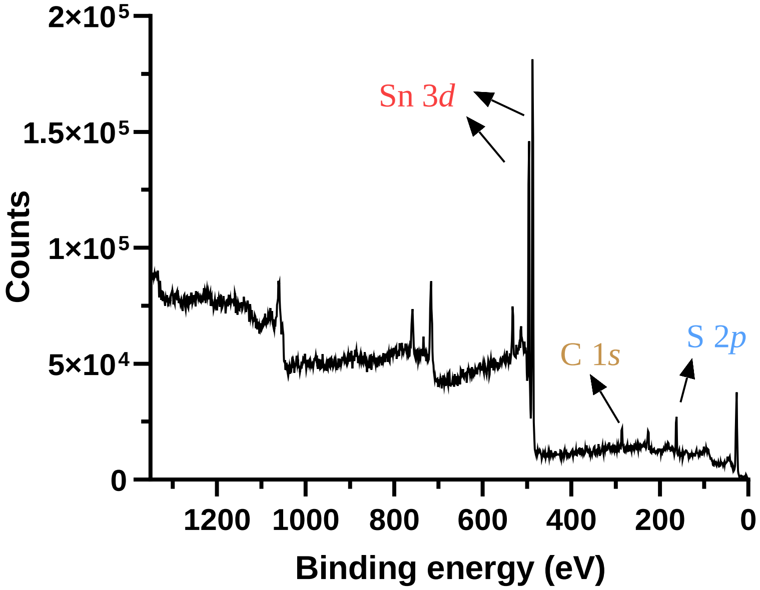

Figure S2. XPS survey spectrum of SnSNPs.

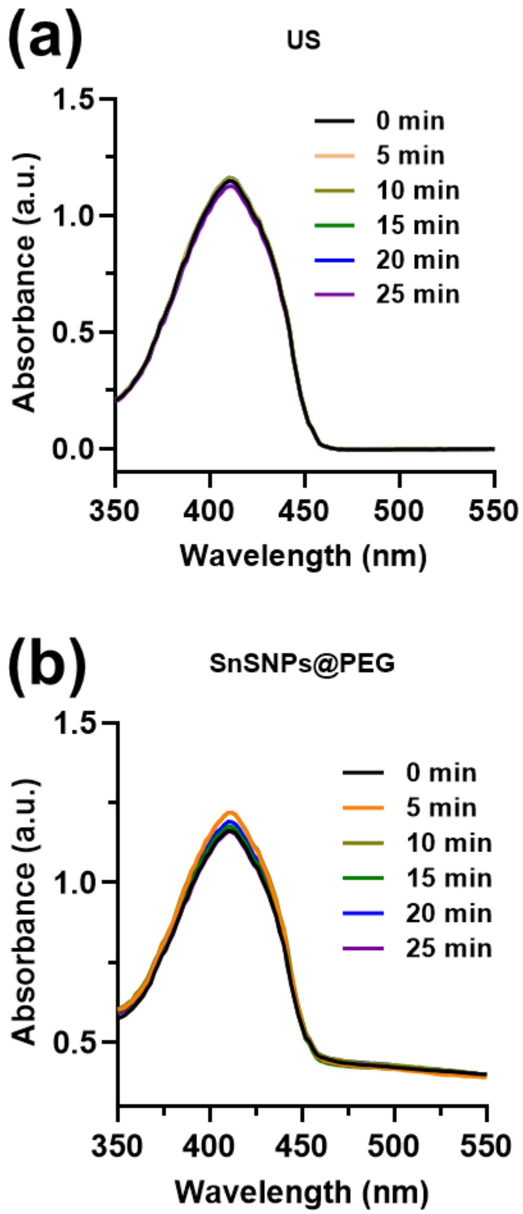

Figure S3. UV−Vis absorbance spectra of the time-dependent oxidation of DPBF (80 μg mL^{-1} in ethanol) by the following treatment: (a) US (1 MHz, 1 W cm^{-2}, 50% duty cycle) only, and (b) SnSNPs@PEG (200 μg mL^{-1}) only.

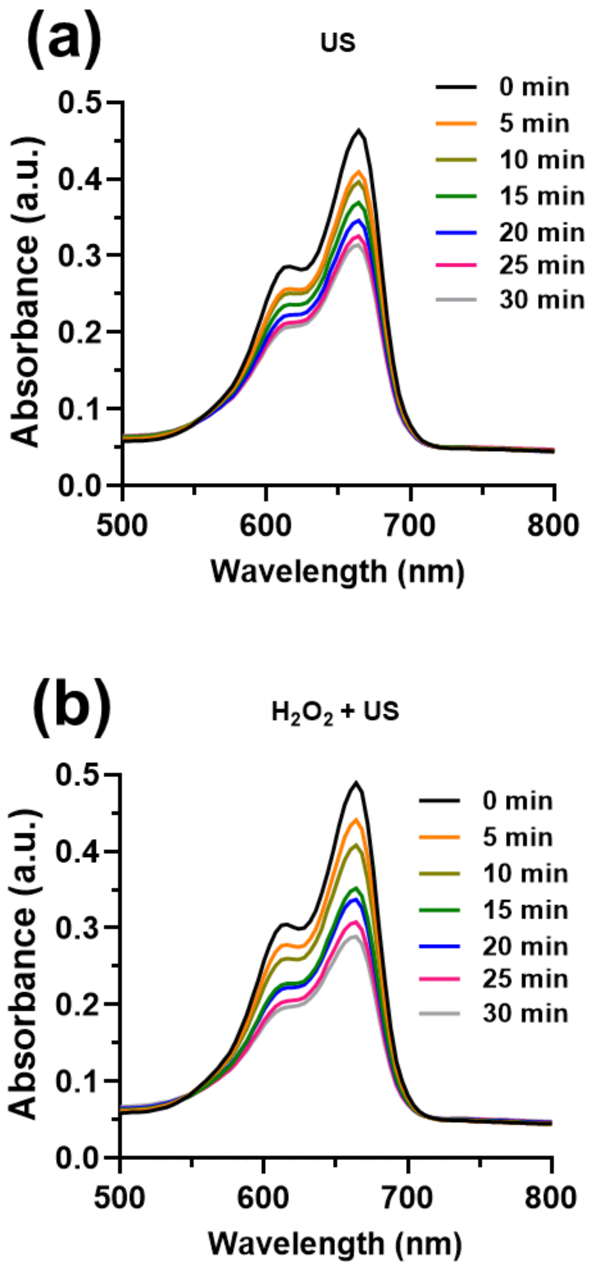

Figure S4. UV−Vis absorbance spectra of the time-dependent oxidation of MB (5 μg mL^{-1} in PBS) by the following treatment: (a) US (1 MHz, 2 W cm^{-2}, 50% duty cycle) only, and (b) H_2O_2 (50 μM) + US.

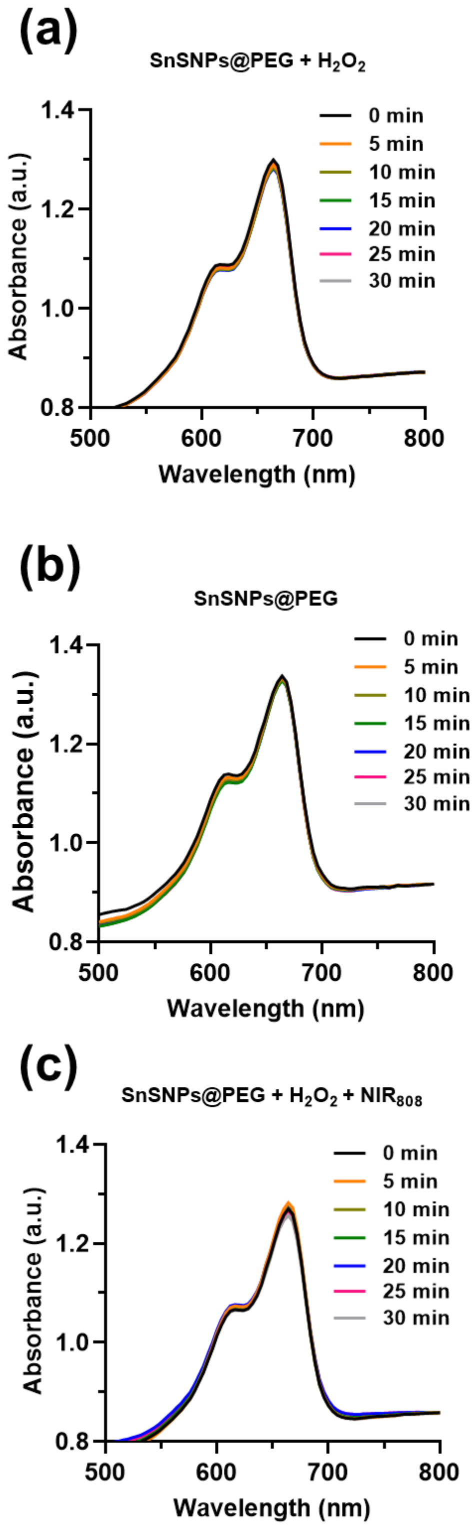

Figure S5. UV−Vis absorbance spectra of the time-dependent oxidation of MB (5 μg mL^{-1} in PBS) by the following treatment: (a) SnSNPs@PEG (100 μg mL^{-1}) + H_2O_2 (50 μM), (b) SnSNPs@PEG only, and (c) SnSNPs@PEG + H_2O_2 + NIR_{808} (1.0 W cm^{-2}).

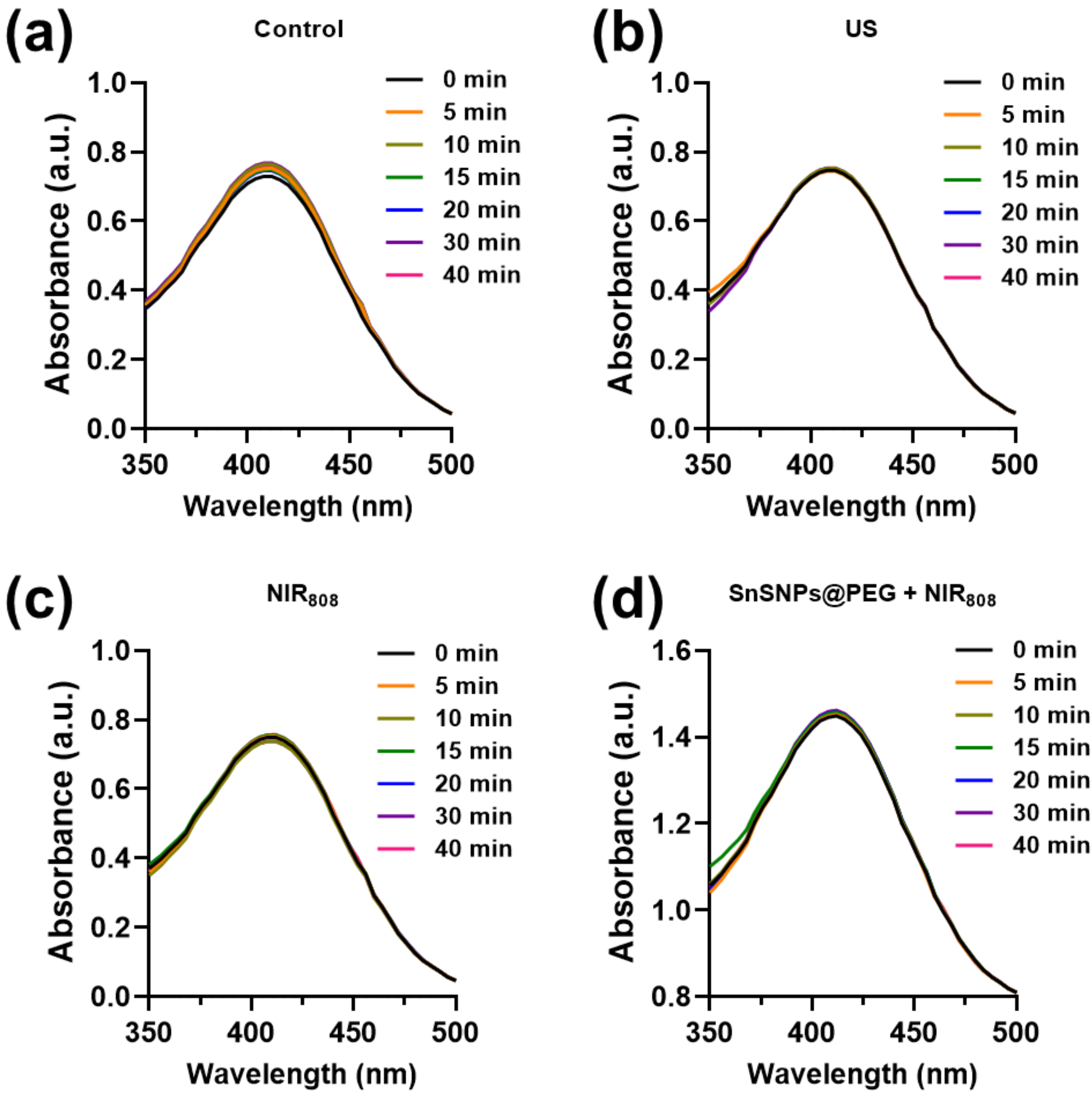

Figure S6. UV−Vis absorbance spectra of the time-dependent oxidation of GSH (30 μg mL^{-1} in PBS) by the following treatment: (a) SnSNPs@PEG (200 μg mL^{-1}), (b) US (1 MHz, 2 W cm^{-2}, 50% duty cycle) only, (c) GSH + NIR_{808} and (d) SnSNPs@PEG + NIR_{808} (1.0 W cm^{-2}).

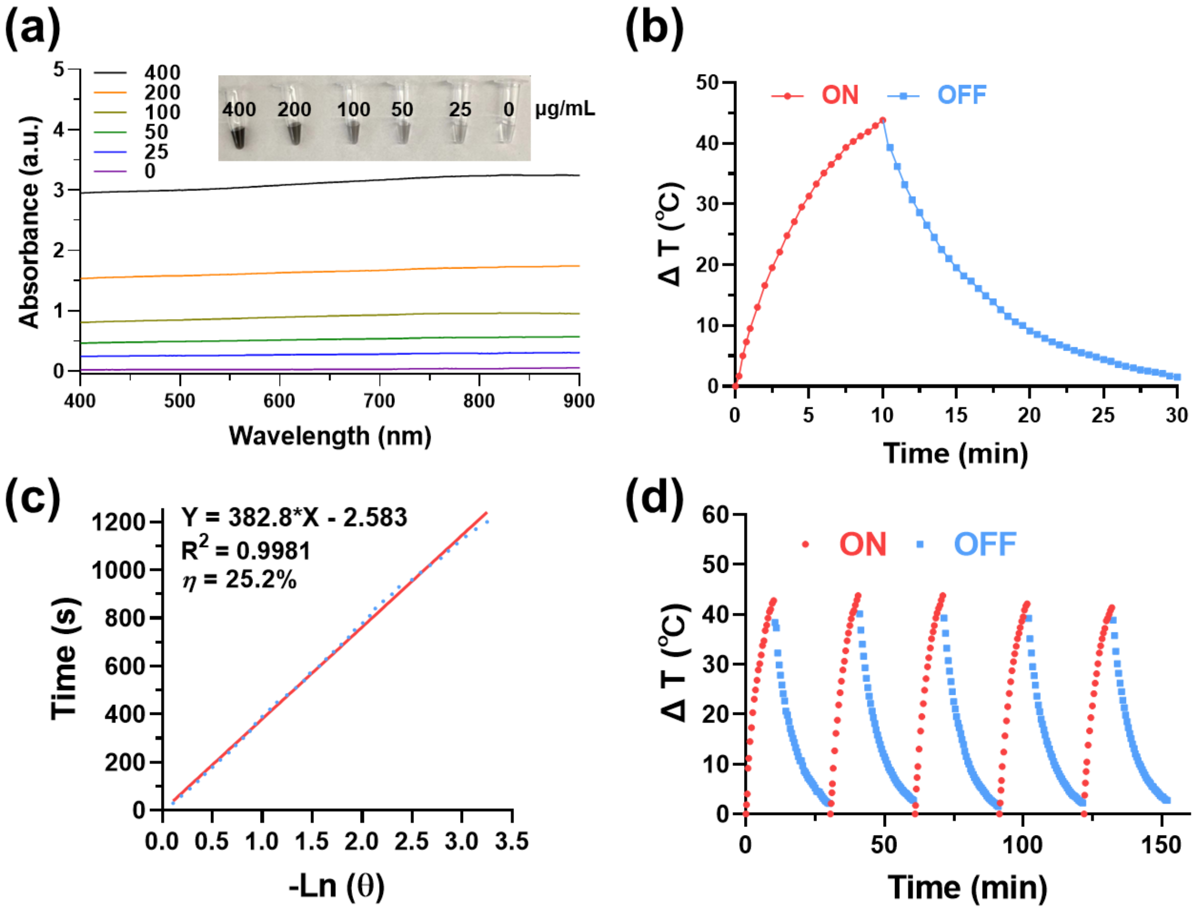

Figure S7. Photothermal performance of SnSNPs@PEG. a) UV-Vis-NIR absorption spectra of SnSNPs@PEG at different concentrations. Inset photo shows SnSNPs@PEG water suspension with the corresponding concentration in μg mL^{-1}. b) Heating and cooling profiles of SnSNPs@PEG (200 μg mL^{-1}) irradiated by an 808 nm NIR laser at the power density of 2.0 W cm^{-2}. c) Liner fitting curve of time and -Ln (θ) from the cooling profile in (b). d) Repetitive five continues heating and cooling profiles of SnSNPs@PEG (200 μg mL^{-1}) irradiated by an 808 nm NIR laser at the power density of 2.0 W cm^{-2}.

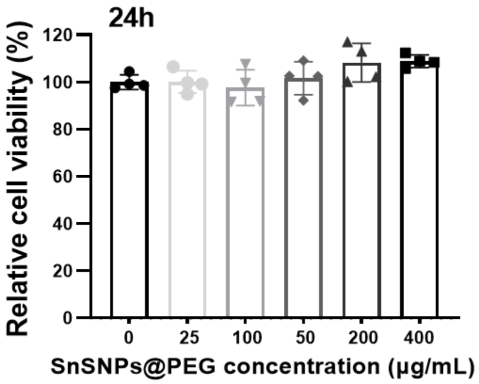

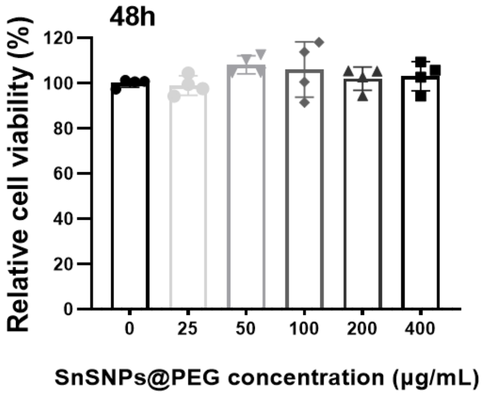

Figure S8. Relative viability of 4T1 cells after incubation with different concentrations of SnSNPs@PEG for 24 and 48 h. Data are from independent samples and are presented as mean ± SD (n = 4).

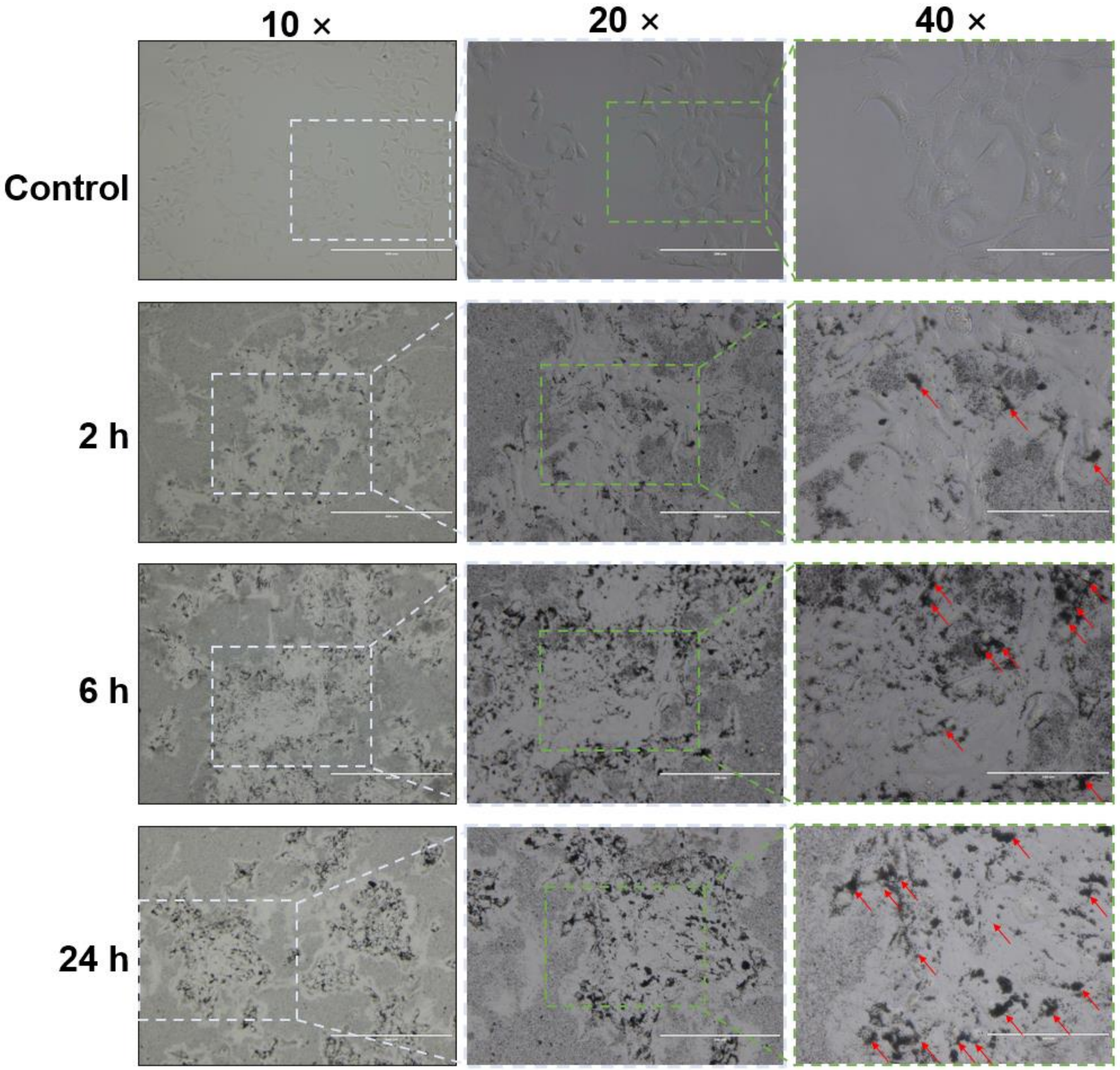

Figure S9. Bright-field microscope images showing time-dependent cellular uptake of SnSNPs@PEG by 4T1 cells. The substantial uptake was highlighted by red arrows. No cytotoxic effect was observed on 4T1 cells given that the substantial cellular uptake of SnSNPs@PEG was observed. Scale bar: 10× = 400 μm, 20× = 200 μm, 40× = 100 μm.

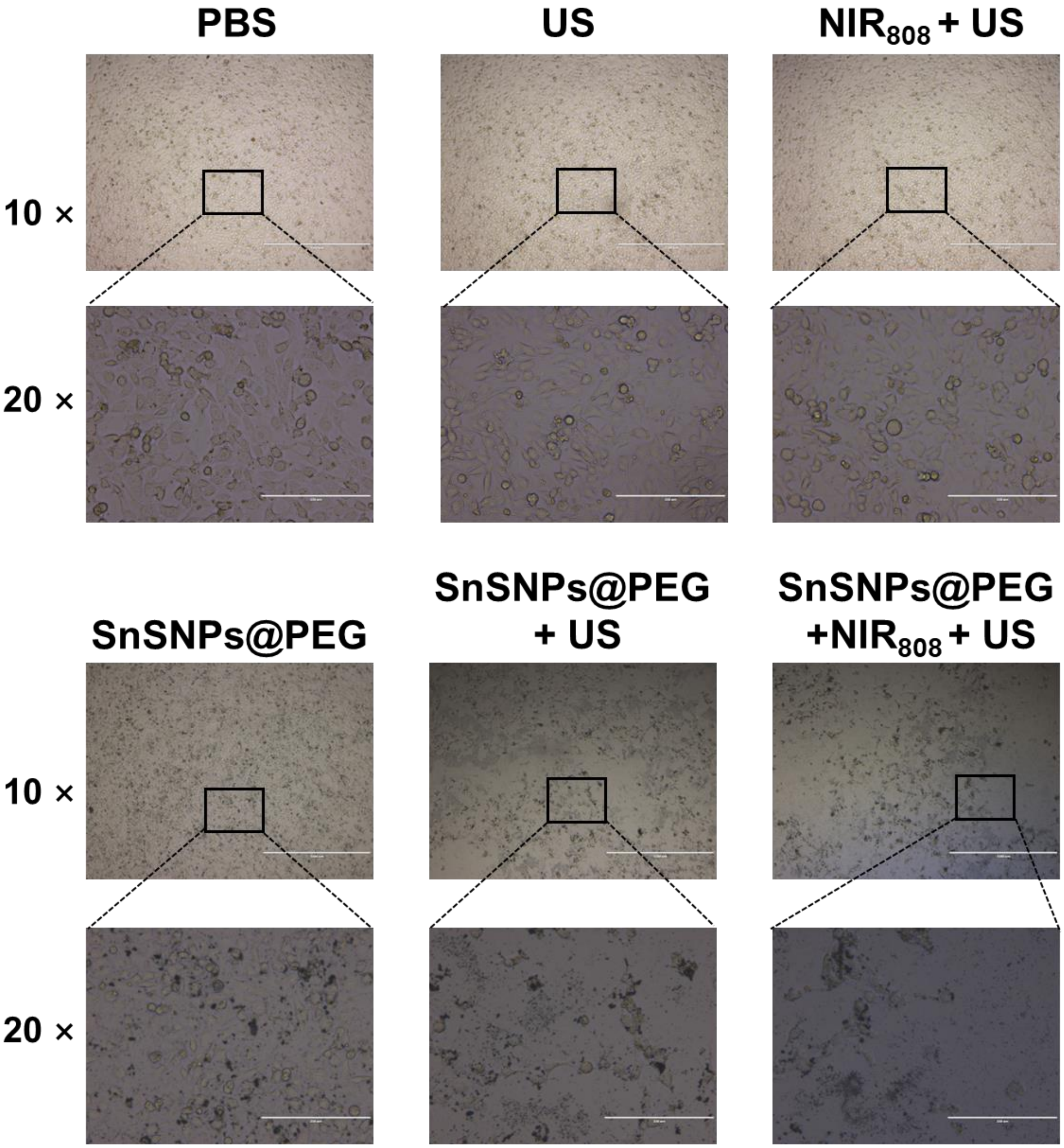

Figure S10. Bright-field microscope images showing 4T1 cells after various treatments. SnSNPs@PEG (200 μg mL^{-1}) with US irradiation (1 MHz, 0.3 W cm^{-2}, 50% duty cycle) or with NIR_{808} (1.0 W cm^{-2}) + US irradiation showed substantial cytotoxic effects. Scale bar: 10 × = 400 μm, 20× = 200 μm.

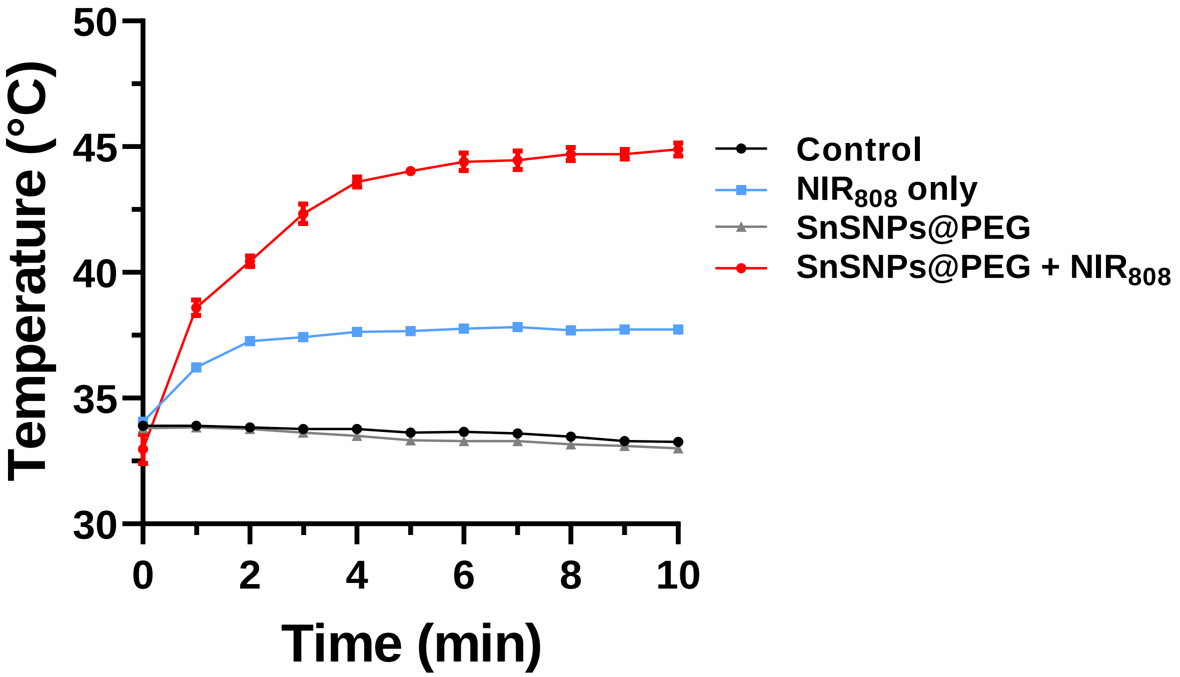

Figure S11. Time-dependent temperature increase profile in 4T1 tumor-bearing mice showing the *in vivo* photothermal effect of SnSNPs@PEG. Temperatures were recorded 12 h after intravenous injection of 100 μL of PBS containing SnSNPs@PEG (10 mg kg^{-1}) under NIR_{808} (1 W cm^{-2}) irradiation. Data are presented as mean ± SD (n = 3).

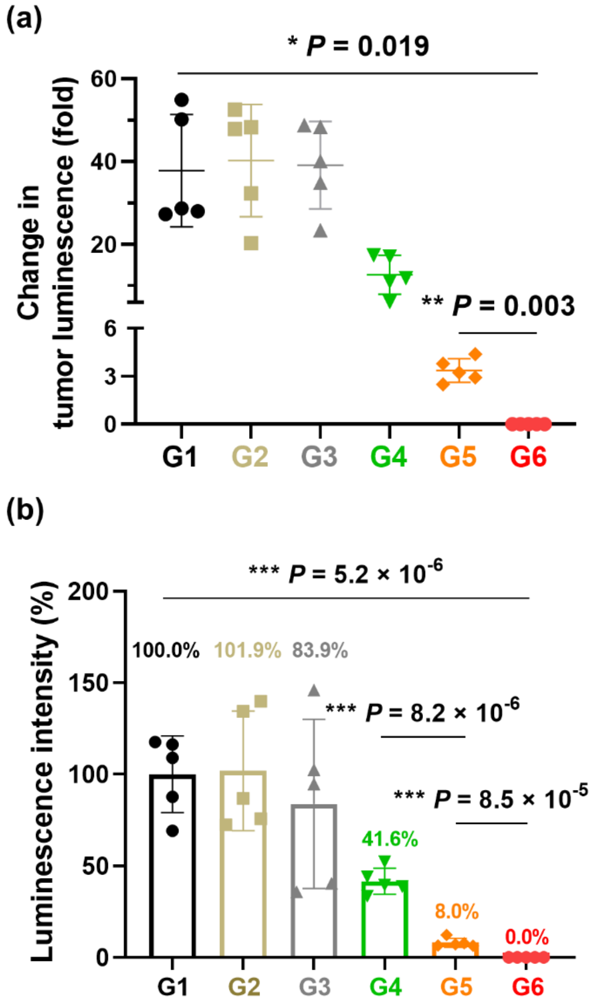

Figure S12. Comparison of luminescence intensity of 4T1 tumor areas after various treatments. (a) Fold-change of tumor luminescence intensity. The intensity change was calculated as luminescence intensity on Day 16/ luminescence intensity on Day 0 (before treatment). (b) Percentage of tumor luminescence intensity. The percentage was calculated as follows: luminescence intensity at Day 16 of each tumor-bearing mice in group/ average luminescence intensity of mice in G1 at Day 16. Statistical analysis between the two groups was performed using two-sided student's *t*-test. ** $P < 0.01$, *** $P < 0.001$. Groups are as follows: control (G1), SnSNPs@PEG (G2), NIR + US (G3), SnSNPs@PEG + US (G4), SnSNPs@PEG + NIR + US (G5), SnSNPs@PEG + NIR + US × 2 (G6), (n = 5).

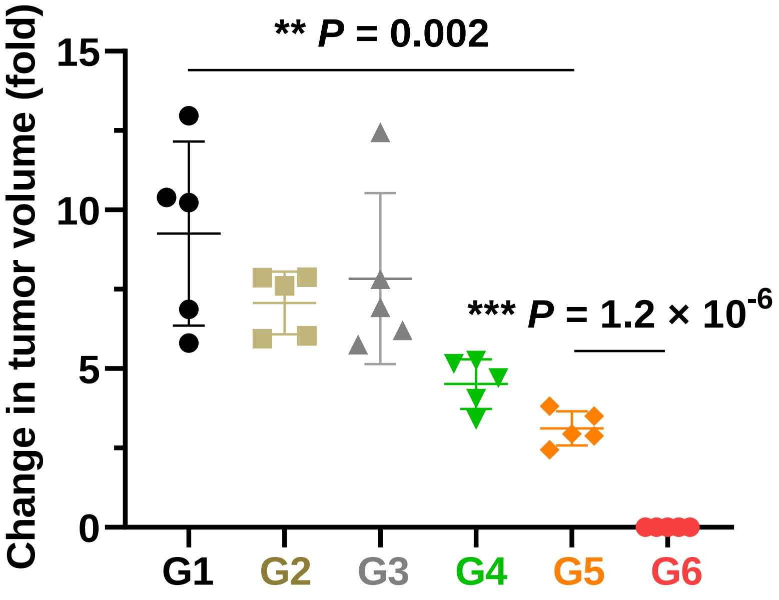

Figure S13. Change in the 4T1 tumor volume after various treatments. The volume change was calculated as follows: tumor volume at Day 16/ tumor volume at Day 0 (before treatment). Data are presented as mean ± SD (n = 5). Statistical analysis between two groups was performed using two-sided student's *t*-test. ** P < 0.01, *** P < 0.001. Groups are as follows: control (G1), SnSNPs@PEG (G2), NIR + US (G3), SnSNPs@PEG + US (G4), SnSNPs@PEG + NIR + US (G5), SnSNPs@PEG + NIR + US × 2 (G6), (n = 5).

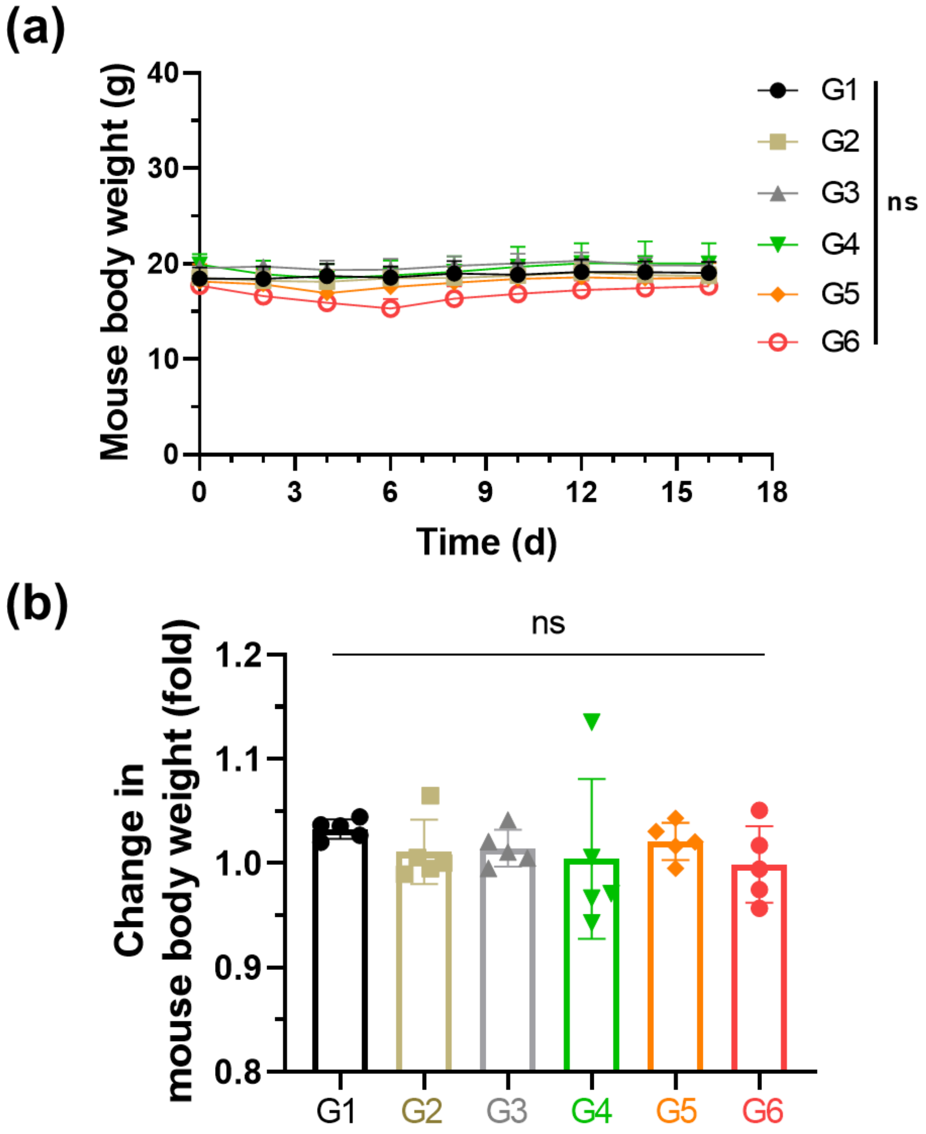

Figure S14. Change in body weight of 4T1 tumor-bearing mice during the SnSNPs@PEG-mediated strategy treatment. (a) Time-dependent body weight of 4T1 tumor-bearing mice of different treatment groups. (n = 5); (b) Change in mouse body weight after various treatments. The mouse body weight change was calculated as follows: mouse body weight at Day 16/ mouse body weight at Day 0 (before treatment). Data are presented as mean ± SD (n = 5). Statistical analysis among groups was performed using one-way ANOVA test. ns, not significant. Groups are as follows: control (G1), SnSNPs@PEG (G2), NIR + US (G3), SnSNPs@PEG + US (G4), SnSNPs@PEG + NIR + US (G5), SnSNPs@PEG + NIR + US × 2 (G6), (n = 5).

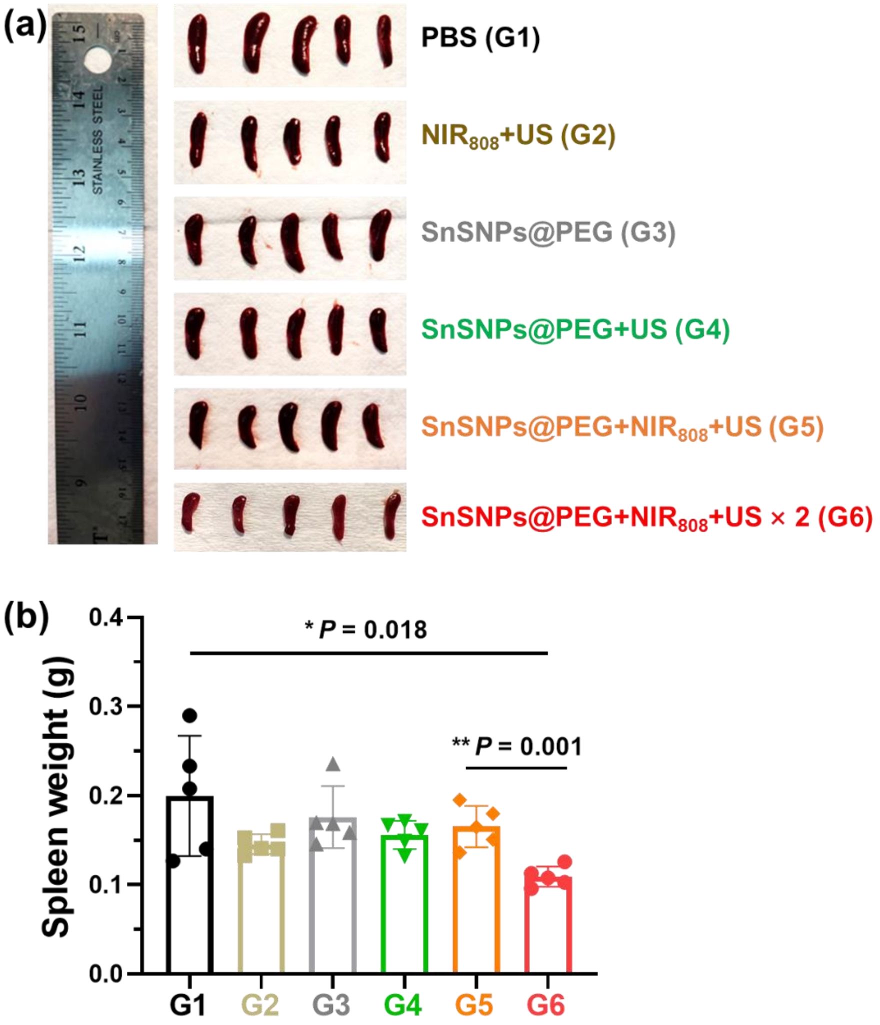

Figure S15. Spleen of 4T1 tumor-bearing mice after SnSNPs@PEG-mediated treatment strategy. (a) Photograph of spleens harvested from 4T1 tumor-bearing mice at Day 16 from different treatment groups (n = 5); b) Spleen weight of 4T1 tumor-bearing mice shown in (a). Data are presented as mean ± SD. (n = 5). Statistical analysis between two groups was performed using student's *t*-test. * $P < 0.05$, ** $P < 0.01$. Groups are as follows: control (G1), SnSNPs@PEG (G2), NIR + US (G3), SnSNPs@PEG + US (G4), SnSNPs@PEG + NIR + US (G5), SnSNPs@PEG + NIR + US × 2 (G6), (n = 5).

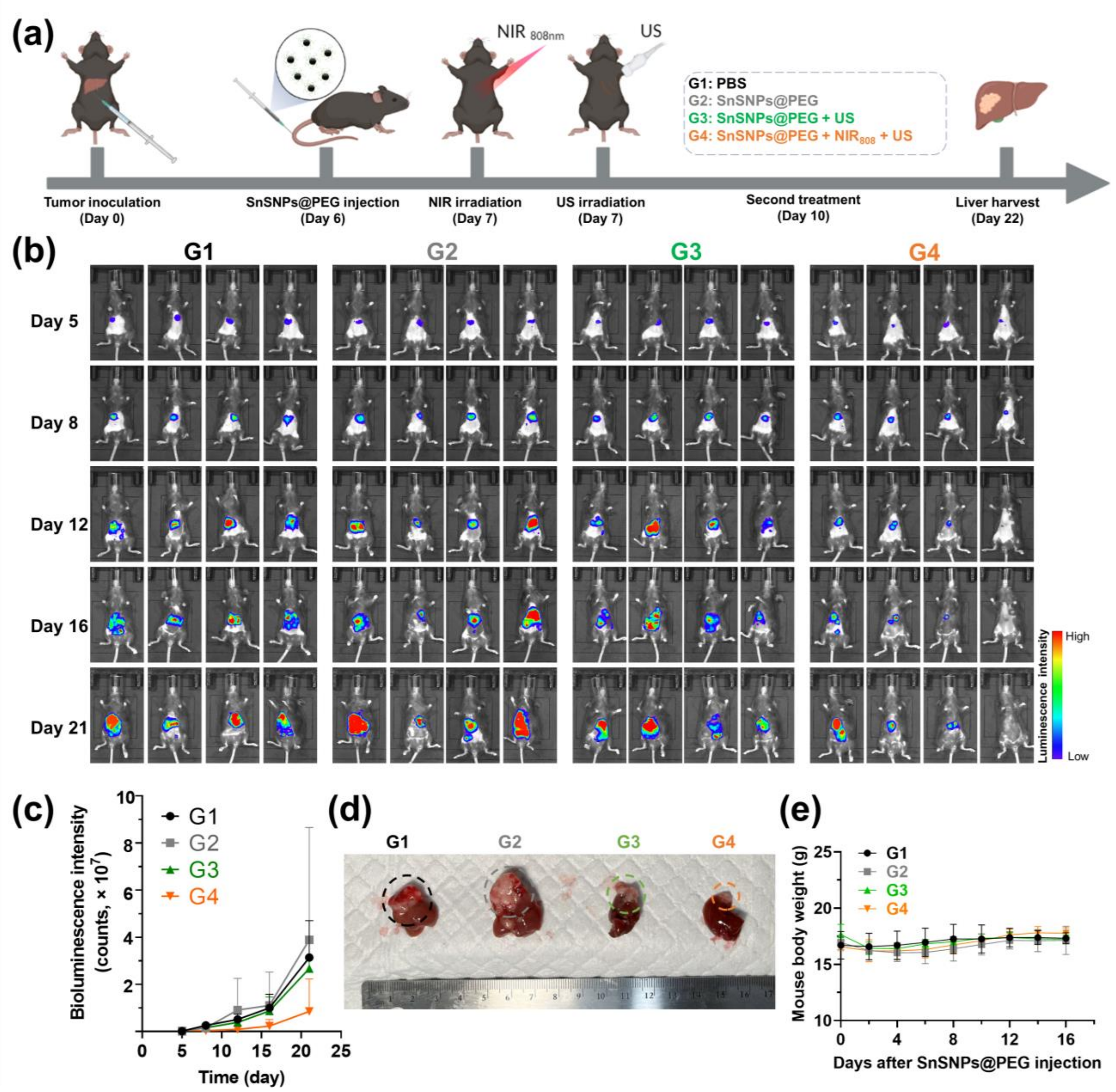

Figure S16. Antitumor efficacy of SnSNPs@PEG-mediated SDT in an orthotopic RIL-175-HCC mouse model. a) Experimental timeline for establishing the RIL-175-HCC mouse model and SnSNPs@PEG-mediated treatment. Illustration was created with BioRender.com. b) Bioluminescence images of orthotopic RIL-175-HCC-bearing mice before, during and after various treatments, including PBS (G1), SnSNPs@PEG (G2), SnSNPs@PEG + US (G3), and SnSNPs@PEG + NIR_{808} + US (G4). The injection volume is 100 μL and the dose of SnSNPs@PEG is 10 mg kg^{-1}. c) Analysis of bioluminescence (counts) of orthotopic RIL-175-HCC-bearing mice before, during and after various treatments. Data are presented as mean ± SD (n = 4). d) Photograph of excised livers with RIL-175-HCC after various treatments (Day 22). Tumor areas are highlighted by circles. e) Time-dependent body weight of RIL-175-HCC-bearing mice after receiving various treatments.

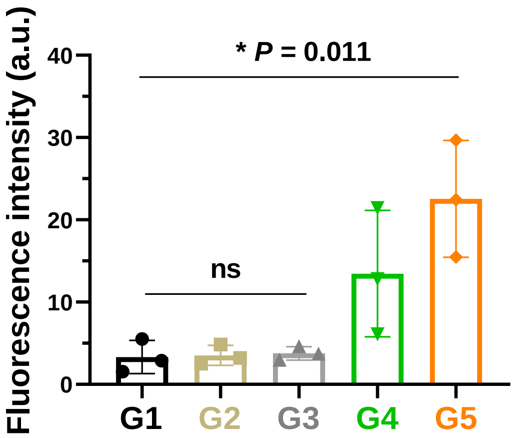

Figure S17. Semi-quantitative analysis of green fluorescence intensity showing the ROS level in tumors of 4T1 tumor-bearing mice after various treatments. Data are presented as mean ± SD (n = 3). Statistical analysis between two groups was performed using student's *t*-test. * $P < 0.05$. Groups are as follows: control (G1), NIR + US (G2), SnSNPs@PEG (G3), SnSNPs@PEG + US (G4), SnSNPs@PEG + NIR + US (G5), (n = 3).

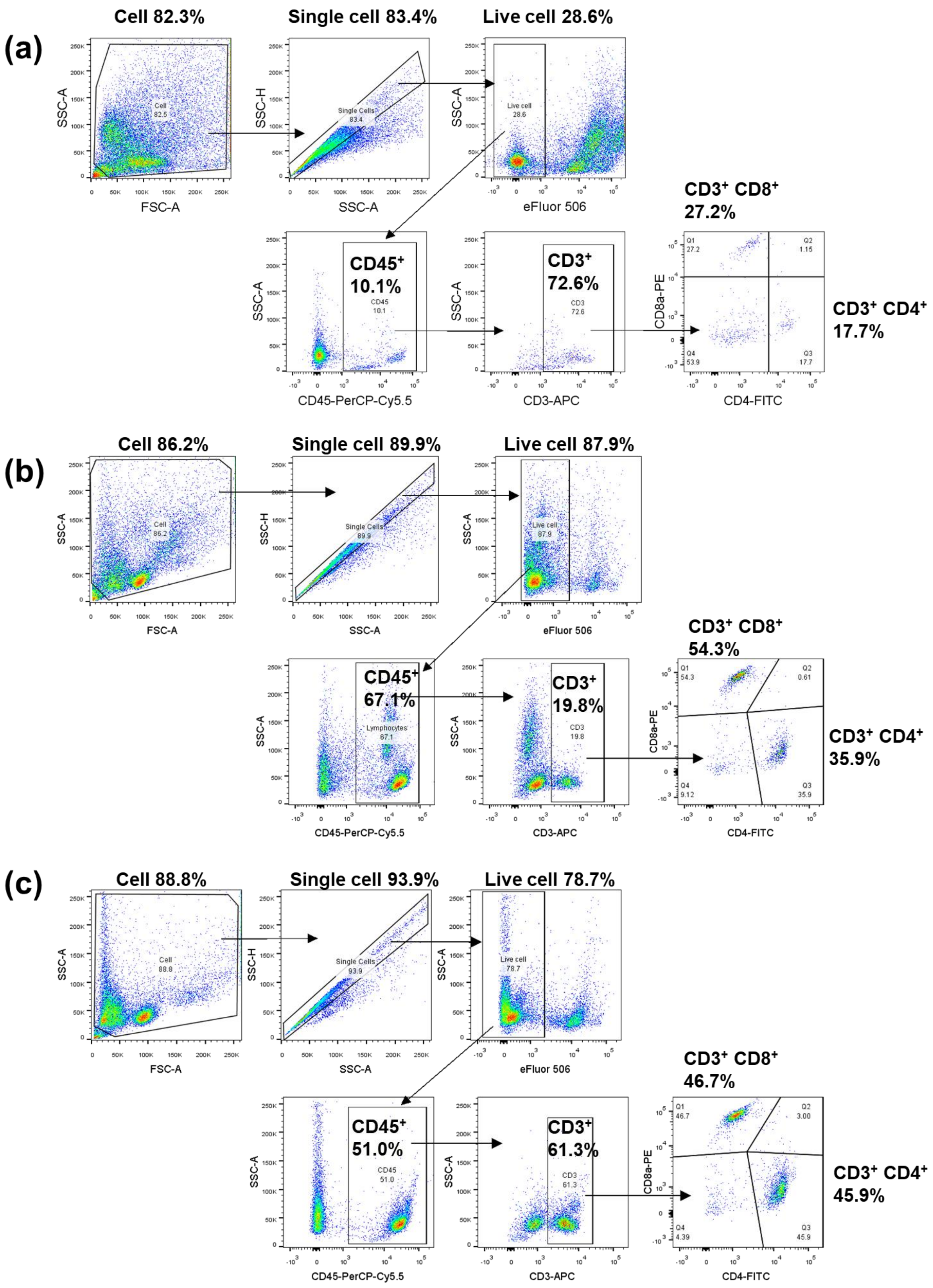
(a)
Cell 82.3%
Single cell 83.4%
Live cell 28.6%
SSC-A
FSC-A
SSC-H
SSC-A
eFluor 506
Cell 82.5
Single Cells 83.4
Live cell 28.6
CD45⁺ 10.1%
CD45 10.1
CD45-PerCP-Cy5.5
CD3⁺ 72.6%
CD3 72.6
CD3-APC
CD3⁺ CD8⁺ 27.2%
CD3⁺ CD4⁺ 17.7%
Q1 27.2
Q2 1.15
Q3 17.7
Q4 53.9
CD8a-PE
CD4-FITC
(b)
Cell 86.2%
Single cell 89.9%
Live cell 87.9%
Cell 86.2
Single Cells 89.9
Live cell 87.9
CD45⁺ 67.1%
Lymphocytes 67.1
CD3⁺ 19.8%
CD3 19.8
CD3⁺ CD8⁺ 54.3%
CD3⁺ CD4⁺ 35.9%
Q1 54.3
Q2 0.61
Q3 35.9
Q4 9.12
(c)
Cell 88.8%
Single cell 93.9%
Live cell 78.7%
Cell 88.8
Single Cells 93.9
Live cell 78.7
CD45⁺ 51.0%
CD45 51.0
CD3⁺ 61.3%
CD3 61.3
CD3⁺ CD8⁺ 46.7%
CD3⁺ CD4⁺ 45.9%
Q1 46.7
Q2 3.00
Q3 45.9
Q4 4.39

Figure S18. Gating strategies to identify $CD45^+CD3^+CD4^+$ and $CD45^+CD3^+CD8^+$ T lymphocytes in the tumor, spleen and lymph nodes of 4T1 tumor-bearing mice following the SnSNPs@PEG-mediated treatment strategy. (a) tumor, (b) spleen and (c) lymph node. Single live cells were first gated, followed by a selection of $CD45^+CD3^+$ T lymphocytes. Subsequently, the populations of $CD45^+CD3^+CD4^+$ and $CD45^+CD3^+CD8^+$ T lymphocytes were distinguished and analyzed.

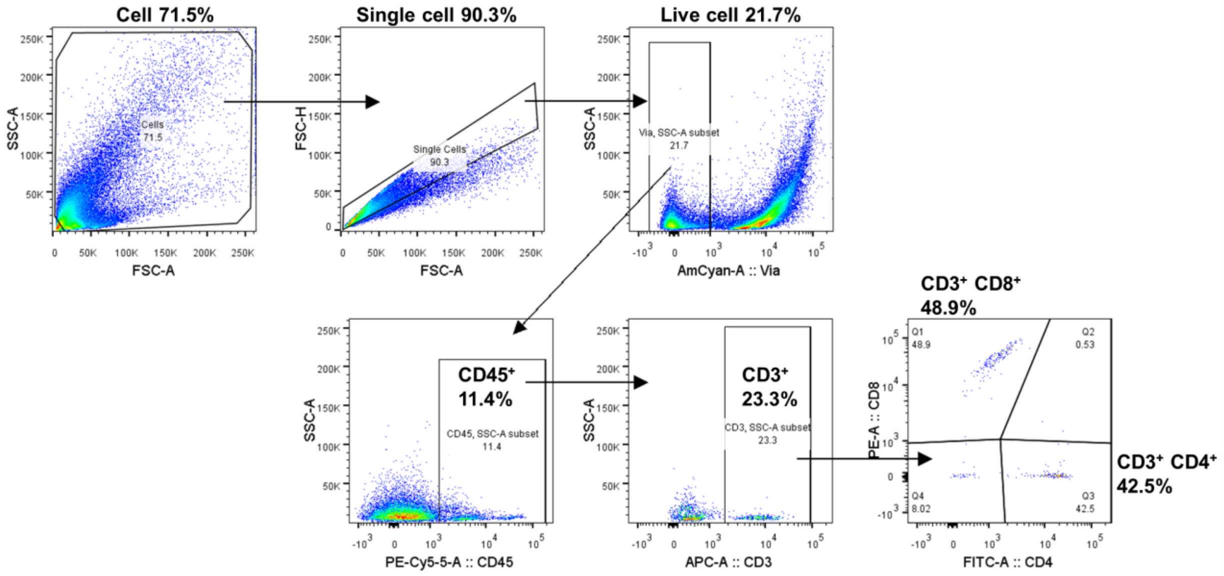

Figure S19. Gating strategy to identify $CD45^+CD3^+CD4^+$ and $CD45^+CD3^+CD8^+$ T lymphocytes in the RIL-175-HCC following the SnSNPs@PEG-mediated treatment strategy. Single live cells were first gated, followed by a selection of $CD45^+CD3^+$ T lymphocytes. Subsequently, the populations of $CD45^+CD3^+CD4^+$ and $CD45^+CD3^+CD8^+$ T lymphocytes were distinguished and analyzed.

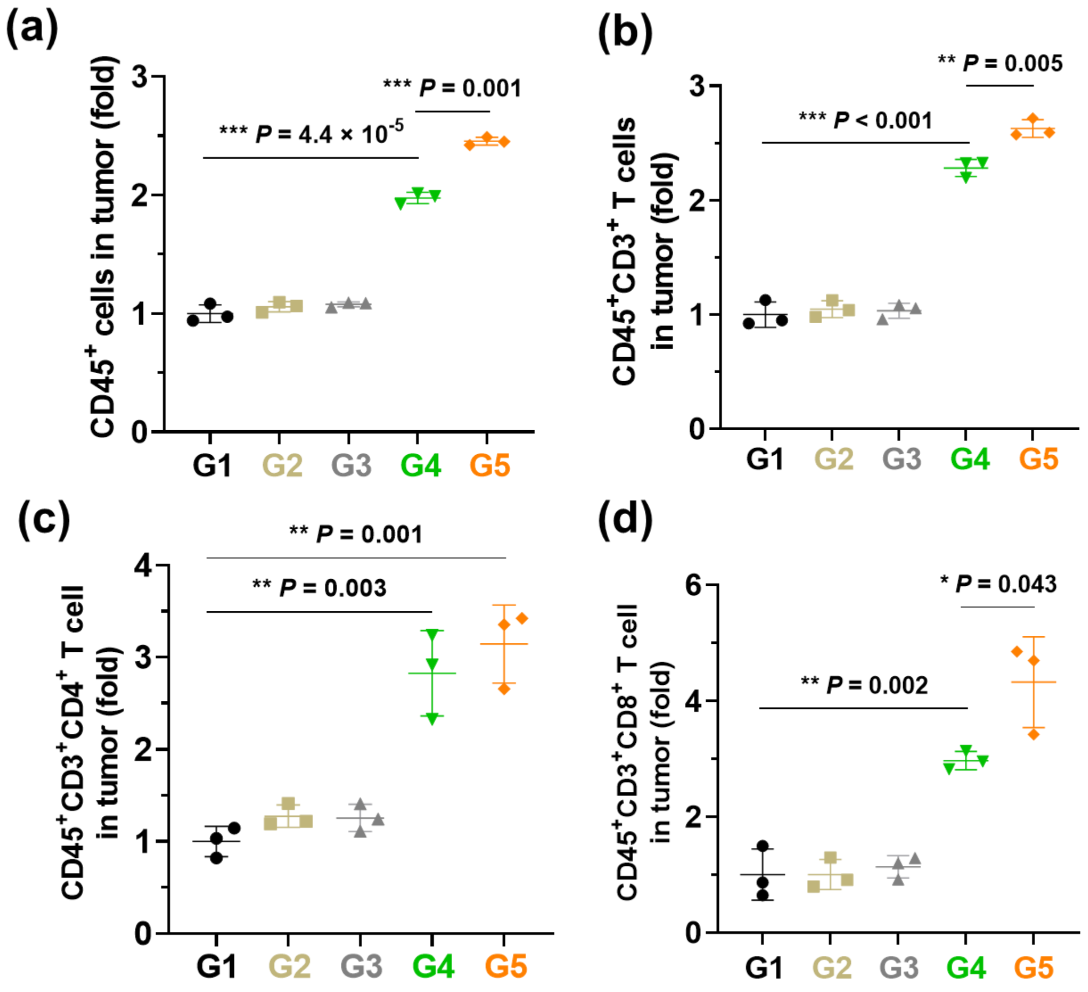

Figure S20. Comparison of immune cell levels in 4T1 tumors in different groups after SnSNPs@PEG-mediated treatment strategy. (a) $CD45^+$ immune cells, (b) $CD45^+CD3^+$ T cells, (c) $CD45^+CD3^+CD4^+$ T cells, and (d) $CD45^+CD3^+CD8^+$ T cells in tumors after various treatments. Data are presented as mean ± SD (n = 3). Statistical analysis between two groups was performed using two-sided student's *t*-test. * $P < 0.05$, ** $P < 0.01$, *** $P < 0.001$. Groups are as follows: control (G1), NIR + US (G2), SnSNPs@PEG (G3), SnSNPs@PEG + US (G4), SnSNPs@PEG + NIR + US (G5), (n = 3).

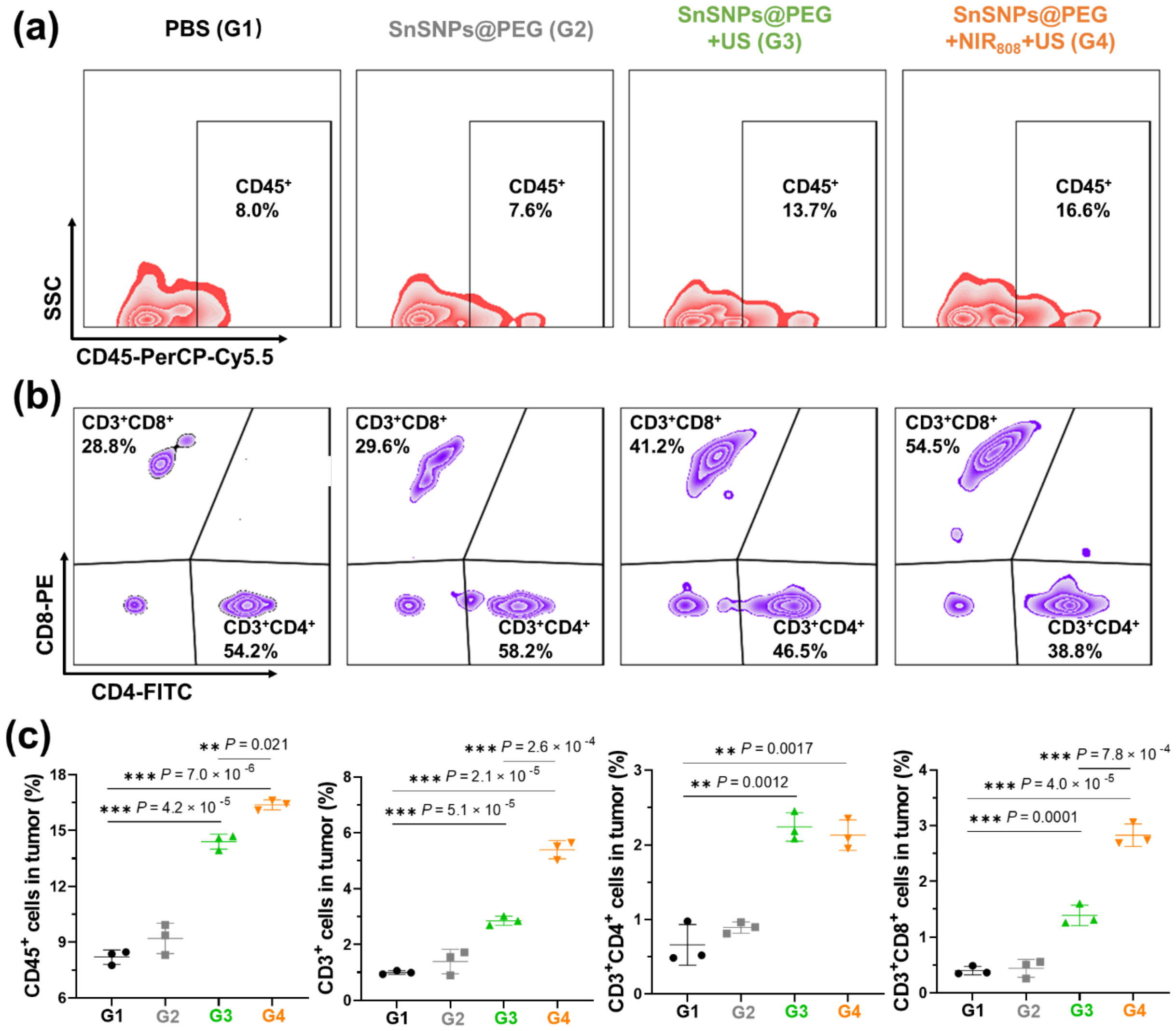

Figure S21. Flow cytometry analysis of (a) $CD45^+$ cells, (b)(c) $CD45^+CD3^+$ lymphocytes, $CD45^+CD3^+CD4^+$ T lymphocytes and $CD45^+CD3^+CD8^+$ T lymphocytes in RIL-175-HCC after different treatments (n = 3). Statistical analysis between two groups was performed using two-sided student's *t*-test. Data are presented as mean ± SD (n = 3) * $P < 0.05$, ** $P < 0.01$, *** $P < 0.001$. Groups are as follows: control (G1), SnSNPs@PEG (G2), SnSNPs@PEG + US (G3), SnSNPs@PEG + NIR + US (G4), (n = 3).

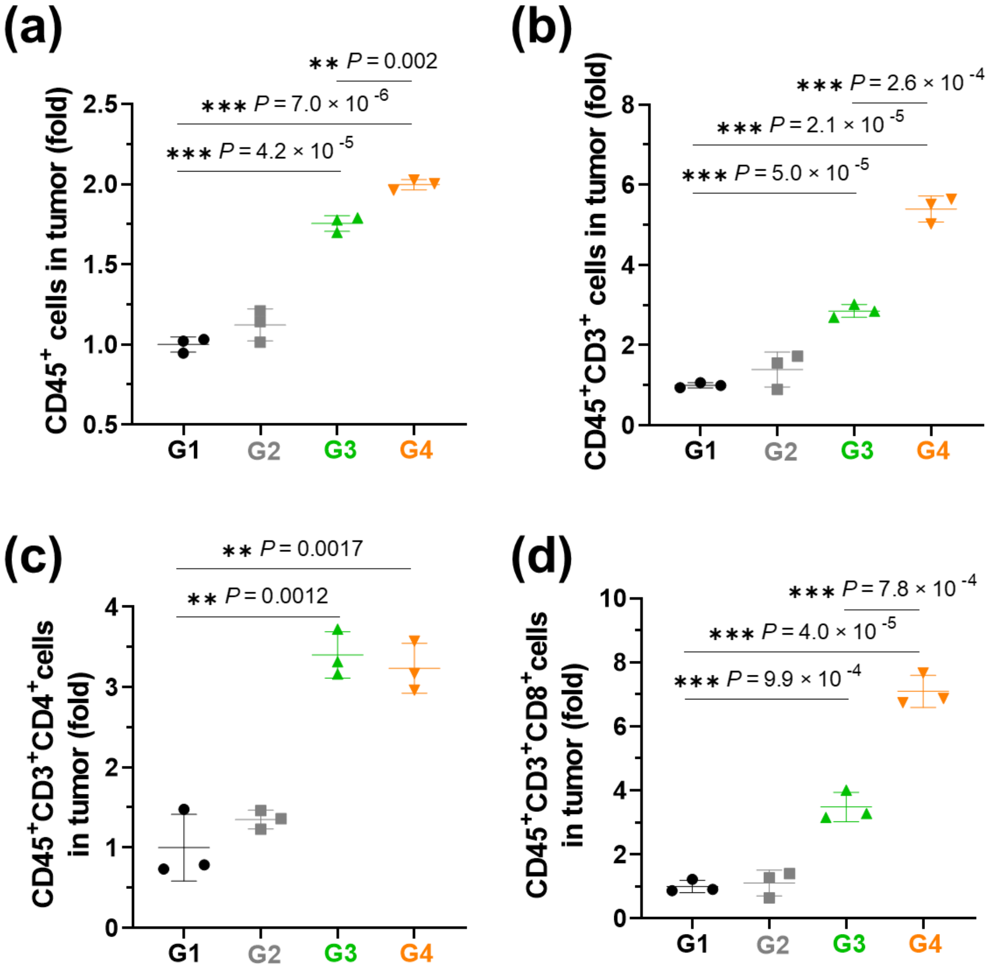

Figure S22. Comparison of the levels of (a) $CD45^+$ immune cells, (b) $CD45^+CD3^+$ T cells, (c) $CD45^+CD3^+CD4^+$ T cells, and (d) $CD45^+CD3^+CD8^+$ T cells in RIL-175-HCC-bearing mice after various treatments (n = 3). Statistical analysis between two groups was performed using two-sided student's *t*-test. Data are presented as mean ± SD (n = 3). * $P < 0.05$, ** $P < 0.01$, *** $P < 0.001$. Groups are as follows: control (G1), SnSNPs@PEG (G2), SnSNPs@PEG + US (G3), SnSNPs@PEG + NIR + US (G4), (n = 3).

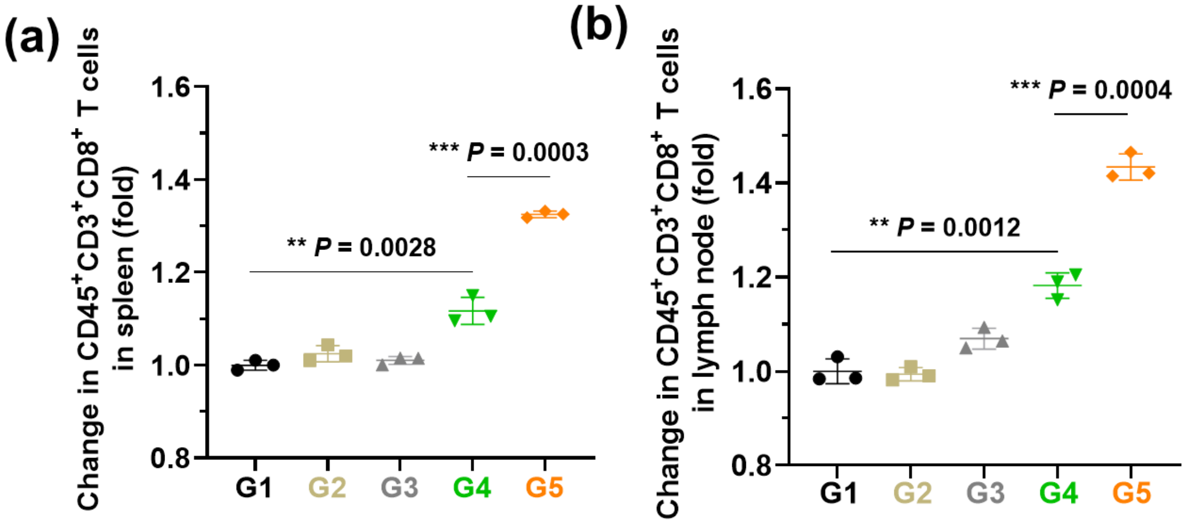

Figure S23. Comparison of $CD45^+CD3^+CD8^+$ T cell levels in spleens and lymph nodes of 4T1 tumor-bearing mice in different groups after SnSNPs@PEG-mediated treatment strategy. (a) spleens and (b) lymph nodes of tumor-bearing mice after various treatments. Statistical analysis between two groups was performed using two-sided student's *t*-test. Data are presented as mean ± SD (n = 3) * $P < 0.05$, ** $P < 0.01$, *** $P < 0.001$. Groups are as follows: control (G1), NIR + US (G2), SnSNPs@PEG (G3), SnSNPs@PEG + US (G4), SnSNPs@PEG + NIR + US (G5), (n = 3).

Table S1. Comparison of the bandgap of SnSNPs with other nano-sonosensitizers.

Sonosensitizer	*Structure*	*Reported bandgap*	*Reference*
SnS	Nanoparticle	1.18 eV	This work
$Bi@BiO_{2-x}@Bi_2S_3$	Nanoparticle	1.43 eV	[1]
α-$Fe_2O_3@Pt$	Heterostructure particle	1.83 eV	[2]
WOx	Nanobelt	2.11 eV	[3]
$Ti(Oi\text{-}Pr)_4@Ag$	Metal−organic framework	2.11 eV	[4]
Sn	Nanosheet	2.3 eV	[5]
$BiVO_4$	Nanorod	2.5 eV	[6]
Vanadium carbide	Carbon dot	2.57 eV	[7]
Sodium molybdenum bronze	Nanoparticle	2.7 eV	[8]
$TiH_{1.924}$	Nanodot	2.7 eV	[9]
TiO_2	Nanoparticle	3.2 eV	[10]

Table S2. Antibody used for the flow cytometry analysis of T cells.

**Antibody**	**Brand and Catalog #**	**Concentration for use**
TruStain FcX™ (anti-mouse CD16/32)	Miltenyi Biotec; 130-092-575	1.0 µg/mL
PerCP/Cyanine5.5 anti-mouse CD45	BioLegend; 109828	2.0 µg/mL
APC anti-mouse CD3	BioLegend; 100236	2.5 µg/mL
FITC anti-mouse CD4	BioLegend; 100406	1.5 µg/mL
PE anti-mouse CD8	BioLegend; 100708	1.5 µg/mL

Reference

1. Song, K., *et al.* Biodegradable Bismuth-Based Nano-Heterojunction for Enhanced Sonodynamic Oncotherapy through Charge Separation Engineering. *Adv. Healthc. Mater.* **11**, e2102503 (2022).
2. Zhang, T., *et al.* α-Fe_2O_3@Pt heterostructure particles to enable sonodynamic therapy with self-supplied O_2 and imaging-guidance. *J. Nanobiotechnology* **19**, 358 (2021).
3. Zhou, Y., *et al.* Oxygen-Deficient Tungsten Oxide (WO_x) Nanobelts with pH-Sensitive Degradation for Enhanced Sonodynamic Therapy of Cancer. *ACS Nano* **16**, 17242-17256 (2022).
4. Meng, X., *et al.* Ag-Doped Metal-Organic Frameworks' Heterostructure for Sonodynamic Therapy of Deep-Seated Cancer and Bacterial Infection. *ACS Nano* **17**, 1174–1186 (2023).
5. Chen, W., *et al.* Stanene-Based Nanosheets for β-Elemene Delivery and Ultrasound-Mediated Combination Cancer Therapy. *Angew. Chem. Int. Ed.* **60**, 7155-7164 (2021).
6. Yang, Z., *et al.* Conferring $BiVO_4$ Nanorods with Oxygen Vacancies to Realize Enhanced Sonodynamic Cancer Therapy. *Angew. Chem. Int. Ed.* **61**, e202209484 (2022).
7. Wang, H., *et al.* A MXene-derived redox homeostasis regulator perturbs the Nrf2 antioxidant program for reinforced sonodynamic therapy. *Chem. Sci.* **13**, 6704-6714 (2022).
8. He, X., *et al.* NIR-II photo-amplified sonodynamic therapy using sodium molybdenum bronze nanoplatform against subcutaneous Staphylococcus aureus infection. *Adv. Funct. Mater.* **32**, 2203964 (2022).
9. Gong, F., *et al.* Preparation of $TiH_{1.924}$ nanodots by liquid-phase exfoliation for enhanced sonodynamic cancer therapy. *Nat. Commun.* **11**, 3712 (2020).
10. George, S., *et al.* Role of Fe doping in tuning the band gap of TiO_2 for the photo-oxidation-induced cytotoxicity paradigm. *J. Am. Chem. Soc.* **133**, 11270-11278 (2011).
