# Supplementary material for: Nanosensitizer-mediated augmentation of sonodynamic therapy efficacy and antitumor immunity
Source: Nat Commun. 2023 Nov 1;14:6973. doi: 10.1038/s41467-023-42509-7 (PMC10620173; doi:10.1038/s41467-023-42509-7)
Supplement: Supplementary file 3 — Reporting Summary [file 41467_2023_42509_MOESM3_ESM.pdf]

nature portfolio

Corresponding author(s): Tian Xie, Wei Tao

Last updated by author(s): Sep 7, 2023

Reporting Summary

Nature Portfolio wishes to improve the reproducibility of the work that we publish. This form provides structure for consistency and transparency in reporting. For further information on Nature Portfolio policies, see our Editorial Policies and the Editorial Policy Checklist.

Statistics

For all statistical analyses, confirm that the following items are present in the figure legend, table legend, main text, or Methods section.

n/a	Confirmed	
☐	☒	The exact sample size (*n*) for each experimental group/condition, given as a discrete number and unit of measurement
☐	☒	A statement on whether measurements were taken from distinct samples or whether the same sample was measured repeatedly
☐	☒	The statistical test(s) used AND whether they are one- or two-sided<br>*Only common tests should be described solely by name; describe more complex techniques in the Methods section.*
☒	☐	A description of all covariates tested
☒	☐	A description of any assumptions or corrections, such as tests of normality and adjustment for multiple comparisons
☐	☒	A full description of the statistical parameters including central tendency (e.g. means) or other basic estimates (e.g. regression coefficient) AND variation (e.g. standard deviation) or associated estimates of uncertainty (e.g. confidence intervals)
☐	☒	For null hypothesis testing, the test statistic (e.g. *F*, *t*, *r*) with confidence intervals, effect sizes, degrees of freedom and *P* value noted<br>*Give P values as exact values whenever suitable.*
☒	☐	For Bayesian analysis, information on the choice of priors and Markov chain Monte Carlo settings
☐	☒	For hierarchical and complex designs, identification of the appropriate level for tests and full reporting of outcomes
☒	☐	Estimates of effect sizes (e.g. Cohen's *d*, Pearson's *r*), indicating how they were calculated

Our web collection on statistics for biologists contains articles on many of the points above.

Software and code

Policy information about availability of computer code

Data collection

BD LSR II was used to collect flow cytometry data. TEM data were collected using Tecnai G2 F20 (FEI, Netherlands). The hydrodynamic sizes were measured using a ZetaPALS (Brookhaven Instruments). The diffuse reflectance spectrum was measured by a UV–Vis–NIR spectrophotometer (Shimadzu UV-3600i Plus, Japan). X-ray photoelectron spectroscopy was measured by ESCALAB 250Xi (Thermo Scientific). X-ray diffraction pattern was measured by MiniFlex600 diffractometer (Rigaku, Japan). Fourier transform infrared spectrometry was measured by Nicolet iS50 FT-IR Spectrometer (Thermo Fisher Scientific, USA). Thermogravimetric data was obtained by Synchronous DSC-TGA Q600 Thermogravimetric Analyzer (TA Instruments, USA). Thermal images and temperature were collected using T100 IR thermal camera (Fluke, USA). Fluorescence cell images was performed on Axio Vert A1 (Zesis, Germany). Confocol fluorescence cell imaging was performed on FV1000 microscope (Olympus, Japan). Bright field cell morphology was observed by a XL core system microscope (EVOS, USA). Cell viability data was collected using Infinite M200PRO plate reader (Tecan, Swiss). In vivo biodistribution fluorescence images were obtained by imaging system (PXi 4 Touch, Syngene). Luminescence images were collected and quantified using IVIS imaging system (Lumina LT Series III, PerkinElmer, USA). The concentration of Sn was determined by the High Resolution (magnetic sector field)-Inductively coupled plasma mass spectrometry (HR-ICP-MS) (Nu Instruments AttoM ES).

Data analysis

Analysis of flow cytometry data was performed with FlowJo v10.7 software. GraphPad Prism Version 9.0 was used for data statistics and statistical significance calculation. Fluorescence images were analyzed using FluoView Software and ZEN 3.6 software (blue edition). Microsoft Excel was used for tumor size analysis. Biodistribution images was analyzed using Fiji. Images of the tissue sections were analyzed using CaseViewer 3.0.

For manuscripts utilizing custom algorithms or software that are central to the research but not yet described in published literature, software must be made available to editors and reviewers. We strongly encourage code deposition in a community repository (e.g. GitHub). See the Nature Portfolio guidelines for submitting code & software for further information.

Data

Policy information about availability of data

All manuscripts must include a data availability statement. This statement should provide the following information, where applicable:

- Accession codes, unique identifiers, or web links for publicly available datasets
- A description of any restrictions on data availability
- For clinical datasets or third party data, please ensure that the statement adheres to our policy

All study data are available within this manuscript and the associated Supplementary Information. Other data are available from the corresponding author upon reasonable request. Source data are provided with this paper.

Human research participants

Policy information about studies involving human research participants and Sex and Gender in Research.

Reporting on sex and gender	N/A
Population characteristics	N/A
Recruitment	N/A
Ethics oversight	N/A

Note that full information on the approval of the study protocol must also be provided in the manuscript.

Field-specific reporting

Please select the one below that is the best fit for your research. If you are not sure, read the appropriate sections before making your selection.

☒ Life sciences ☐ Behavioural & social sciences ☐ Ecological, evolutionary & environmental sciences

For a reference copy of the document with all sections, see nature.com/documents/nr-reporting-summary-flat.pdf

Life sciences study design

All studies must disclose on these points even when the disclosure is negative.

Sample size	Sample sizes for animal experiments were not calculated prior to performing experiments. The number of animals in each group was determined according to previous studies cited in our manuscript. The size of each sample is in close agreement with those studies already published and with the need for statistical analysis to discuss the degree of differences and measure the variability of these in vivo data.
Data exclusions	No data were excluded from the analysis.
Replication	All the experimental findings were replicated with the number of replicates (at least two independent experiments), animals and variation shown by n and SD . All experimental findings were replicated successfully using biological replicates on different days.
Randomization	Randomization was performed only for in vivo studies. FL imaging study: the tumor-bearing BALB/c mice were distributed randomly into different groups. Therapeutics study: the BALB/c and C57BL/6J mice were inoculate with tumor cell suspensions and after randomly distributed into the control and experimental groups. Each specific treatment was administrated to animals according to established schedules and regimens.
Blinding	Investigators were blinded when grouping tumor-bearing mice, measuring tumor size, body weight, imaging biodistribution and luminescence. Investigators for TEM characterization were blinded to the samples. Blinding was not possible for other experiments because it was impossible for hiding the labeling from the experimenter or sample analyzer.

Reporting for specific materials, systems and methods

We require information from authors about some types of materials, experimental systems and methods used in many studies. Here, indicate whether each material, system or method listed is relevant to your study. If you are not sure if a list item applies to your research, read the appropriate section before selecting a response.

Materials & experimental systems

n/a	Involved in the study
☐	☒ Antibodies
☐	☒ Eukaryotic cell lines
☒	☐ Palaeontology and archaeology
☐	☒ Animals and other organisms
☒	☐ Clinical data
☒	☐ Dual use research of concern

Methods

n/a	Involved in the study
☒	☐ ChIP-seq
☐	☒ Flow cytometry
☒	☐ MRI-based neuroimaging

Antibodies

Antibodies used

Flow cytometry:
anti-CD16/32 antibody, mouse (Cat# 130-092-575, Miltenyi Biotec, 0.5 μg/mL);
https://www.miltenyibiotec.com/US-en/products/fcr-blocking-reagent-mouse.html#gref

anti-CD45-PerCP-Cy5.5, mouse (Clone 104, Cat# 109828, Biolegend, 2.0 μg/mL);
https://www.biolegend.com/nl-be/products/percp-cyanine5-5-anti-mouse-cd452-antibody-4271

anti-CD3-APC, mouse (Clone 17A2, Cat# 100236, Biolegend, 2.5 μg/mL);
https://www.biolegend.com/en-us/products/apc-anti-mouse-cd3-antibody-8055

anti-CD4-FITC, mouse (Clone GK1.5, Cat# 100406, Biolegend, 1.5 μg/mL);
https://www.biolegend.com/nl-be/products/fitc-anti-mouse-cd4-antibody-248

anti-CD8a-PE, mouse (Clone 53-6.7, Cat# 100708, Biolegend, 1.5 μg/mL) antibodies.
https://www.biolegend.com/nl-be/products/pe-anti-mouse-cd8a-antibody-155

Validation

All the above are well characterized commercial antibodies. For each one, the specificity has been tested by the manufacturer and verified independently by previous published studies. Validation profiles and relevant citations can be found in the links provided.

Eukaryotic cell lines

Policy information about cell lines and Sex and Gender in Research

Cell line source(s)

Mouse triple-negative breast cancer cell line (4T1, CRL-2539) and the luciferase-expressing cell line (4T1-luc2, CRL-2539-Luc2) were obtained from the American Type Culture Collections (ATCC). The murine hepatocellular carcinoma (HCC) cell line RIL-175-luc was obtained from Prof. Dan G. Duda's lab at Massachusetts General Hospital, Boston, USA.

Authentication

ATCC used morphology, karyotyping, and PCR based approaches to confirm the identity of cell lines and to rule out both intra- and interspecies contamination. Also, the cell lines were frequently checked by their morphological features under microscope.

Mycoplasma contamination

All cells were negative for mycoplasma.

Commonly misidentified lines
(See ICLAC register)

No commonly misidentified cell lines were used.

Animals and other research organisms

Policy information about studies involving animals; ARRIVE guidelines recommended for reporting animal research, and Sex and Gender in Research

Laboratory animals

Six-to-eight week-old female BALB/c and C57BL/6J mice were purchased from the Jackson Laboratories and housed in a pathogen-free animal facility of Brigham and Women's Hospital. All animals were housed in individually ventilated cage with 12-hour alternate light and dark cycles and at controlled ambient temperature (68-79 F) with humidity between 30% and 70%.

Wild animals

No wild animals were used in this study.

Reporting on sex

Female.

Field-collected samples

This study did not involve samples collected from the fields.

Ethics oversight

All animal experiments were carried out with review and approval from the Institutional Animal Care and Use Committees of Brigham and Women's Hospital and are in compliance with federal and state laws.

Note that full information on the approval of the study protocol must also be provided in the manuscript.

Flow Cytometry

Plots

Confirm that:

☒ The axis labels state the marker and fluorochrome used (e.g. CD4-FITC).

☒ The axis scales are clearly visible. Include numbers along axes only for bottom left plot of group (a 'group' is an analysis of identical markers).

☒ All plots are contour plots with outliers or pseudocolor plots.

☒ A numerical value for number of cells or percentage (with statistics) is provided.

Methodology

Sample preparation	Sections of the Experimental Methods (Enhanced SDT induces anti-tumor immunity) contain details of sample preparation, including preparation of single-cell suspensions derived from tumors, spleens and lymph nodes from tumor-bearing mice and staining protocols.
Instrument	The BD LSR II
Software	FlowJo v10.7.
Cell population abundance	N/A
Gating strategy	Generally, cells were first gated to exclude debris (using FSC-A vs. SSC-A). Singlet cells were gated using SSC-A versus SSC-H and then viable cells were gated using the fixable viability dye. Positive cell populations were determined by the specific antibodies and the cell populations within the gate were further analyzed based on expression of specific markers.

☒ Tick this box to confirm that a figure exemplifying the gating strategy is provided in the Supplementary Information.
